# Assessing process, content, and politics in developing the global health sector strategy on sexually transmitted infections 2016–2021: Implementation opportunities for policymakers

**DOI:** 10.1371/journal.pmed.1002330

**Published:** 2017-06-27

**Authors:** Andy Seale, Nathalie Broutet, Manjulaa Narasimhan

**Affiliations:** World Health Organization, Geneva, Switzerland

## Abstract

Andrew Seale and colleagues discuss the development of a global strategy to counter sexually transmitted infections.

**Summary points**

- Sexually transmitted infections (STIs) present significant health and economic challenges in all countries and yet are rarely prioritised for coordinated strategic attention.
- The 2016 World Health Assembly adopted a global health sector strategy on STIs for 2016–2021, including ambitious 2020 and 2030 goals aligned with broader sustainable development goals and targets of ending disease epidemics as public health concerns by 2030.
- The strategy requires actions at the country level, guided and led by governments, supported by the World Health Organization (WHO) and other partners.
- A number of barriers frustrate efforts to take the response to STIs to scale, including insufficient incidence data and disease surveillance, and political resistance to scientifically-proven and often cost-effective interventions and approaches.
- Country-level success in strategy implementation requires that WHO, Ministries of Health, and broader stakeholders look beyond the interventions required for effective STI management to also consider the broader context, processes, and politics of STIs when building and strengthening responses.
- Evaluating progress towards the strategy’s 2020 coverage targets and 2030 coverage and impact targets will be the key success-measurement tool, yet limiting analysis to the frame of coverage and impact targets alone will deny important opportunities to drive action and to evolve policies and programmes in dynamic contexts.
- Exploring and assessing the implementation context, political interest, and potential of health policies can ensure early identification of challenges and opportunities when focused on national-level policy uptake and execution.
- This paper applies an analytical approach to the global strategy that includes an investigation of 3 domains: process, programmatic, and political. Key questions are proposed to guide exploration of these domains to help identify and address barriers to, and leverage solutions for, policy success.

## Introduction

In May 2016, the World Health Assembly (WHA) adopted a global health sector strategy on sexually transmitted infections (STIs) for 2016–2021 [1] that outlines 2020 and 2030 targets and builds on what was learned from implementing the Global Strategy for Prevention and Control of Sexually Transmitted Infections: 2006–2015 [2,3]. The new strategy was adopted alongside linked global health sector strategies on HIV and viral hepatitis [4,5].

Beyond HIV, STIs are rarely prioritised for comprehensive action despite a considerable disease burden and clear health and economic arguments [1]. Like other overlooked health issues, a lack of data creates barriers to further action and investment. The new strategy presents a logical structure and clear rationale for action that includes establishing baseline data for key STIs in 2018, against which future progress will be measured. The strategy’s success will be determined by how extensively it is embraced and implemented by partners, and at national and WHO institutional levels.

While defining and measuring a policy’s success requires an evaluation of its uptake and implementation, it can be beneficial to also explore the following: how well the policy’s technical content and goals are grounded in the latest science and evidence; the power dynamics, political, and operational context of the strategy; and the broader policy environment that provides context to the policy [6].

This paper proposes that an exploration of 3 domains, “process, programmatic, and political” [7], can help unpack important stakeholder power dynamics and positions around STI programming that can facilitate or frustrate policy implementation [6].

In the absence of large and well-funded public sector programmes, STI responses are often fragmented, with many uncoordinated service providers from the public, private, and not-for-profit sectors. STI services often fall outside the essential service packages of health financing systems—presenting significant challenges to strategic coordination, equitable service provision, and quality assurance. In addition, STIs, like many other health issues linked to human behaviour, can trigger strong reactions from political, religious, and other cultural commentators. The role of public health professionals is to navigate the politicised and often polarised context of health to ensure that evidence-based policies and programmes are appropriately prioritised and supported [7].

This paper is the first of a series to be published as part of a PLOS Collection, highlighting the importance of strengthening the response to STIs as part of the broader 2030 Agenda for Sustainable Development.

## Analytical approach

While there are no standard evaluation tools available for measuring the potential success of newly adopted global public health policies and strategies, there are numerous frameworks that can help guide implementation and assessment. This paper adapts Marsh and McConnell’s policy assessment framework [8] (Table 1) to help inform both the organisation of this paper and, potentially, guide future assessment and evaluation undertakings at the national level.

**Table 1:** Evaluation questions adapted from the 9 indicators proposed by Marsh and McConnell’s *A framework for establishing policy success*.

**Process Considerations** • Was legitimacy established when designing the strategy development process? • Was there evidence of participatory practice, informed deliberation, and accountability? • What amendments were made during the process? • To what extent is the strategy based on new ideas or policy instruments, and to what extent did it adopt ideas and references from elsewhere? • Was the strategy development process an efficient use of resources? •Were there any positive or negative unintended process consequences?	**Programmatic/Technical Considerations** • Does the strategy build on the 2006–2015 strategy? • Does the strategy propose an efficient use of resources aligned with public health guidance? • Are the key steps required for strategy implementation and evaluation clear? • In relation to whose interests will strategy success be assessed? For example, Member States? WHO? Partners? Affected communities? • Is the strategy aligned with key WHO/public health priorities, including universal health coverage? • Were there any positive or negative unintended programmatic consequences?	**Political Considerations** • To what extent is the strategy “politically popular”? • Did strategy development build political sustainability and gain the support of a sufficient coalition? • Did the strategy help enhance the credibility of the WHA and/or WHO? • What, if any, political compromises or fallout occurred during the process? • To what extent is it possible to isolate and assess the impact of this strategy from other factors, such as other strategies and/or media influences? • Were there any positive or negative unintended political consequences?

We draw on systematic documentation of key steps in the strategy’s development process that comprises meeting reports; concept notes developed for consultations and other events; email correspondence; analysis of news and social media reporting and web coverage; analysis of consultations, including 5 regional consultations and a 2-month public online consultation; correspondence from Geneva-based missions to the WHO Secretariat; official records and reports from the Executive Board and WHA; and written comments from nongovernmental organisations (NGOs) and interest groups, including those with WHO observer status.

## Process considerations

In 2014, when the process to develop a new global strategy for STIs started, the context for vertical, communicable, disease-focused programmes at WHO was considerably challenging. The organisation was focused on the following: WHO reform and making sense of the Millennium Development Goals (MDGs), preparing for the “post-2015 era” to be later elaborated in the 2030 Agenda for Sustainable Development, strengthening health systems and Universal Health Coverage (UHC), and reprofiling the importance of noncommunicable diseases [9]. In addition, WHO had been criticised in the context of a poorly managed West African Ebola outbreak [10].

HIV financing, a key source for also financing other STIs, had been criticised for skewing health systems in many countries [11], and there was a perception among stakeholders that there would be limited political appetite for continued support for “silo-promoting” or disease-focused strategies that did not also help support broader systems-level investments. Earlier in 2014, the WHO HIV department had anticipated the need to ensure that HIV work was well positioned for a more systems-focused post-MDG era and published a discussion paper [11], which described how HIV programmes could evolve in the context of broader health efforts and the 3 critical areas of UHC: providing health services, covering populations, and covering costs.

While the WHO HIV Department and Global Hepatitis Programme were preparing to develop strategies on HIV and viral hepatitis, the WHO Department of Reproductive Health and Research (RHR) was also preparing to develop a follow-up strategy to the *Global Strategy for the Prevention and Control of Sexually Transmitted Infections*: *2006–2015*. Policy leads from the 3 areas assessed the programmatic and political environment and concluded that a more integrated approach was appropriate for HIV, viral hepatitis, and STIs. The departments also recognised the strategic importance of encouraging stronger programmatic linkages across the strategies. Consequently, a decision was made to “package” the 3 as interlinked strategies—applying UHC as an organising framework to promote integration and a systems focus and to help secure political buy-in both inside WHO and with stakeholders. A cross-departmental strategy-development management team was established to coordinate and oversee the development of the overall strategic approach and its 3 disease components.

Amendments to the strategies were organised in stages: an initial draft of each strategy informed by technical experts was used as a basis for external consultation, revised drafts were produced after the external consultation for internal review, drafts were then prepared for sharing at the Executive Board in January 2016, and the final drafts were prepared for the May 2016 WHA.

Early changes focused on ensuring the strategies were well organised and comprehensive, whereas later amendments were more focused on addressing areas that lacked political consensus, for example, relating to key populations and the Trade-Related Aspects of Intellectual Property Rights. Member States also raised concerns during consultation related to the impact of resource constraints on national programmes and the need to achieve a comprehensive approach to STIs.

The cost and resources used in the strategy development process can influence how stakeholders view a policy during its implementation phase—a cost-efficient and clear process is likely to signal an efficient and clear strategy [8]. The development process for the 3 strategies combined was managed over 2 years for a similar amount to that required for developing a single global strategy.

Key costs included those required for a series of regional, public, and expert consultations; consultancy support for coordination and project management; materials production; and modelling to cost out the strategies. Other resources were leveraged from existing sources, including staff time, the use of departmental website pages, and engagement from existing technical and civil society reference groups. In-kind support was also secured from partners including Member States, for example, Brazil and South Africa hosted regional consultations; Brazil, France, Egypt, Myanmar, and Zimbabwe supported a WHA Technical Briefing; and Brazil and France cohosted information sessions for Geneva missions in 2015 [12,13].

## Technical considerations

### Learning from past strategies

Key learning for the 2016–2021 strategy on STIs was leveraged from understanding the progress and challenges from implementing the previous strategy, which is outlined in the progress report *Implementation of the Global Strategy for Prevention and Control of Sexually Transmitted Infections*: *2006–2015* for the WHA [3]. The report drew on epidemiological and modelling data, the results of a rapid survey of national STI programme managers, and outcomes from a 2014 meeting of technical advisors. The report noted that most countries had adopted the previous strategy and helpfully identified a number of key challenges proposed for inclusion in the 2016–2021 strategy, including the following:

- Additional funding for STI prevalence studies, stronger surveillance systems and STI programmes;
- Reprioritisation of STI prevention and support for innovations, including STI vaccines and STI rapid diagnostic tests, and multipurpose prevention technologies to strengthen linkages to broader sexual and reproductive health and rights (SRHR) issues;
- Taking successful, evidence-based STI programmes among key populations to scale;
- Greater collaboration with the NGO sector;
- A critical focus on antimicrobial resistance, particularly in *Neisseria gonorrhoeae*.

The 2016–2021 strategy also draws on agreed language from WHO’s 2004 global reproductive health strategy to propose a comprehensive approach to SRHR that includes improving antenatal, delivery, postpartum, and newborn care; providing high-quality services for family planning, including infertility services; eliminating unsafe abortion; combating STIs, including HIV, reproductive tract infections, cervical cancer, and other gynaecological morbidities; preventing intimate partner violence and sexual- and gender-based violence; and promoting sexual health and human rights [14].

### Indicators for measuring progress

While the WHO HIV department tracks, in real time, both policy uptake and implementation in relation to its normative guidance [15], the 3 global strategies adopted in 2016 will formally report back twice: on midterm progress to the 71st WHA in 2018 and in a final report on progress towards 2020 targets at the 74th WHA in 2021. WHO will compile the WHA reports based on country evaluations.

Tracking and reporting progress is particularly challenging for the STIs strategy because of the lack of key data for STIs, including the lack of global baselines for syphilis and gonorrhoea incidence. In the absence of data, the strategy proposes the following: establishing global incidence baselines by 2018, assessing progress towards service coverage targets for 2020, and measuring impact through assessing incidence trends in 2030 in the context of ambitious targets aligned with the elimination of STIs as public health concerns (Box 1).

**Box 1. 2020 and 2030 targets of the 2016–2021 global health sector strategy on sexually transmitted infections (STIs)**

The targets recognise that a concerted effort to rapidly scale up effective evidence-based interventions and services can achieve the goal of ending STI epidemics as public health concerns by 2030:

**Global targets for 2030**

- 90% reduction of *Treponema pallidum* incidence globally (2018 global baseline);
- 90% reduction in *Neisseria gonorrhoeae* incidence globally (2018 global baseline);
- 50 or fewer cases of congenital syphilis per 100,000 live births in 80% of countries;
- Sustain 90% national coverage and at least 80% in every district (or equivalent administrative unit) in countries with the human papillomavirus vaccine in their national immunisation programme.

**Global targets for 2020**

- 70% of countries to have STI surveillance systems in place that are able to monitor progress towards the relevant targets;
- 70% of countries have at least 95% of pregnant women screened for HIV and/or syphilis, 95% of pregnant women screened for HIV and/or syphilis with free prior and informed consent, 90% of HIV-positive pregnant women receiving effective treatment, and 95% of syphilis-seropositive pregnant women treated with at least 1 dose of intramuscular benzathine penicillin or another effective regimen;
- 70% of key populations for HIV to have access to a full range of services relevant to STIs and HIV, including condoms;
- 70% of countries provide STI services or links to such services in all primary, HIV, reproductive health, family planning, and antenatal and postnatal care services;
- 70% of countries deliver HPV vaccines through the national immunisation programme;
- 70% of countries report on antimicrobial resistance in *N*. *gonorrhoeae*.

While the steps for global-level evaluation and reporting are clear, they are likely to be challenging at regional and national levels at which STIs lack an institutional home, focal point, or champion. Building a sense of national accountability and ownership for building and sustaining progress is critical and will require considerable technical support and resources. Formally reporting back to the WHA will require input from Member States and WHO and should, as far as possible, also encourage inputs from broader stakeholders.

## Political considerations

The global strategy-development stakeholder consultation process was well documented throughout [16]. Member States themselves were appreciative of the strategy development process and there were several opportunities to engage and shape the strategy over a 2-year period. During Executive Board and WHA deliberations, many Member States expressed confidence in the final documents that had been informed by extensive Member State and stakeholder inputs [17]. A number of briefings organised between the January 2016 Executive Board meeting and the May WHA were particularly important to ensuring a sense of ownership among Member States and unanimous adoption of the strategies.

Several speakers at the WHA highlighted the importance of building on signs of political will and interest as the strategy moves to implementation, as noted in an intervention made by the International Planned Parenthood Federation (IPPF):

*A renewed global focus on key infections*, *such as syphilis*, *gonorrhea*, *and human papillomavirus*, *has the tremendous potential to mitigate the often-hidden impact on still birth*, *cervical cancer*, *HIV transmission*, *and infertility worldwide*. *For these ambitious strategies to become a reality*, *it requires political support*, *financial investment*, *and integrating with existing health systems*, *including community-based service providers*. *We call on the Member States gathered here to show the leadership that is required in all fora*, *including at the High-Level Meeting on Ending AIDS in New York in June*.*—*IPPF statement to the 69^th^ WHA [18].

Given the high level of competing health issues and WHA agenda items, “political popularity” [8] for the 3 strategies was highlighted by the fact that they were adopted unanimously by the 194 WHA Member States, with 45 Member States taking the opportunity to express endorsement for the 3 strategies, with several speaking on behalf of large geographical regions, including the African region. In addition, 11 partner organisations and observers spoke in their favour. Nevertheless, it is important to note that the strategy on STIs was adopted without amendment in part because of the inclusion of text in each of the strategies that recognises “country context” and “country realities” and the late inclusion of a small amendment to the Draft Resolution to recognise national legislative contexts. This inclusion of new language suggests that further country-level multistakeholder consultations should be encouraged around emerging national-level strategies.

A number of issues that WHO maintains are evidence-based yet, to some countries, are politically challenging, include those related to the following: the provision of comprehensive sexuality education, the recognition of sexual and reproductive health and rights, and meeting the sexual health needs of “specific populations” including men who have sex with men and transgendered people as well as other groups such as prisoners, people who use drugs, and mobile populations.

The term “sexual and reproductive health and rights” was proposed in the strategy and, despite opposition to its inclusion from some Member States, there was no request during WHA proceedings to open up the document for amendment, and the terminology remains intact. This term may well be further debated at the national level as national STI policies are developed.

The global strategy calls for countries to explicitly address the needs of a number of named population groups, ensure that laws and policies that promote human rights and gender equality are in place, and ensure that steps are taken to address legal, regulatory, and policy barriers that encourage stigma, discrimination, and violence. In some countries, the key implementation challenges may not be related to weak health systems and the strengthening laboratory or surveillance infrastructure but may require work at the political and policy levels to create an environment that facilitates strategy implementation.

Marsh and McConnell encourage policymakers to explore positive, negative, and neutral unintended consequences that may arise during policy development and implementation [8,19]. An unintended, yet positive, unanticipated process consequence occurred when Member States used the Executive Board and WHA discussions to focus global attention on the shortage of benzathine penicillin used to treat syphilis—generating visibility and further support to this critical health issue.

## Discussion

At different moments during strategy implementation it will be necessary to pull focus on different challenges, opportunities, or bottlenecks that may be characterised as either political, process, or technical in nature. For example, volatile, hostile, or apathetic political contexts might require the prioritisation of work at a political level.

Exploring the global strategy development process with the use of questions adapted from the Marsh and McConnell framework helps highlight several critical learning points for future STI strategy development processes at both global and national levels:

- **Process:** Deliberately and systematically documenting and monitoring strategy-related process and discourse should be a core and budgeted function of the team responsible for STIs. Optimal implementation requires an institutional home that connects policies with planning, research, political, and implementation functions [20].
- **Technical:** The STI field is politicised and technically complex—ensuring that STI work remains evidence-based and supports and reinforces broader health policy goals, for example, UHC, tackling drug resistance, and the elimination of mother-to-child transmission of diseases, offers important opportunities for building stakeholder engagement and ownership. Strong public health policies should also include a focus and reflectivity on monitoring for, and responding to, unintended consequences [19].
- **Political:** Systematically assessing political interest and/or resistance among key internal and external stakeholders can helpfully inform the strategy or policy development process. Adopting management approaches that are encouraged to appreciate power differentials, partnerships, and systems [21] and engage affected communities during policy development and implementation [22] offer opportunities for success.

## Conclusion

The global strategy’s 2020 coverage targets and 2030 coverage and impact targets provide a strong framework for evaluating progress towards eliminating STIs as public health concerns. Yet, given that the context for this work is both politicised and highly dynamic, it is important that evaluation looks beyond coverage and impact targets, otherwise important opportunities to drive comprehensive action and to evolve policies to changing contexts may be overlooked. This paper proposes that expanded assessment includes deliberate analysis of the political context and policy process alongside assessment of progress towards targets and the extent to which technical priorities have been taken on board. The questions proposed in Box 1 can be easily adapted to national-level realities as an additional evaluation and strategy development tool. Both individually and together, exploration of the 3 domains highlighted in this paper can lead to a number of benefits, including increased access to utilisation of quality-integrated services and improved programme efficiency and value for money. Ultimately, it is hoped that these benefits substantially reduce STI incidence as well as improve health, human rights, and quality of life.

## Abbreviations

IPPF: International Planned Parenthood Federation
MDG: Millennium Development Goal
RHR: Reproductive Health and Research
SRHR: sexual and reproductive health and rights
STI: sexually transmitted infection
UHC: Universal Health Coverage
WHA: World Health Assembly
WHO: World Health Organization
